# Supportive Neighborhoods, Family Resilience and Flourishing in Childhood and Adolescence

**DOI:** 10.3390/children9040495

**Published:** 2022-04-01

**Authors:** Sheila Barnhart, Molly Bode, Michael C. Gearhart, Kathryn Maguire-Jack

**Affiliations:** 1 College of Social Work, University of Kentucky, Lexington, KY 40506, USA; molly.bode2@uky.edu; 2 School of Social Work, University of Missouri, St. Louis, MO 63121, USA; gearhartm@umsl.edu; 3 School of Social Work, University of Michigan, Ann Arbor, MI 48109, USA; kmjack@umich.edu

**Keywords:** child flourishing, adolescent flourishing, neighborhood social cohesion, physical neighborhood environments, family resilience

## Abstract

Flourishing is linked with health and well-being in childhood and adulthood. This study applied a promotive factors model to examine how neighborhood assets might benefit child and adolescent flourishing by promoting family resilience. Using data from the combined 2018 and 2019 National Survey of Children’s Health, structural equation models tested direct and indirect relationships between neighborhood physical environment, neighborhood social cohesion, family resilience, and flourishing among 18,396 children and 24,817 adolescents. After controlling for multiple covariates that may influence flourishing, the models supported that higher levels of neighborhood social cohesion were directly associated with higher levels of flourishing adolescents, and indirectly by positive associations with family resilience for both children and adolescents. No indirect effects between neighborhood physical environments and flourishing were supported by the data for either children or adolescents. However, neighborhood physical environments were positively associated with adolescent flourishing. Understanding social environmental factors that strengthen and enhance child and adolescent flourishing are critical toward designing prevention, intervention, and policy efforts that can build on the existing strengths of families and their communities.

## 1. Introduction

Over the past two decades, the prevalence of child and adolescents mental health problems continues to increase [1,2]. Identifying and understanding the social environmental factors that promote mental health and flourishing is a necessary and pragmatic step toward assuaging this growing public health concern. Because child and adolescents psychological well-being is significantly linked to family environment [3], and families are nested in communities, it is imperative to understand how the family context and community context can help promote child and family well-being.

Research on child and family resilience traditionally centers on understanding if the presence of protective and promotive factors divert or attenuate (i.e. moderate) the effects of risk(s) on health and developmental outcomes [4,5,6]. Scholarship in this arena often utilize moderation analyses to examine the interplay between intra-personal, inter-personal, and community promotive and protective factors that can be incorporated into designing or enhancing prevention, intervention, and policy efforts to promote optimal outcomes. While these compensatory and protective models of risk and resilience help us understand how children and adolescents yield favorable outcomes by factors that attenuate adversity [5], the direct impact of promotive factors are not often the focus. It is plausible that promotive factors can reach beyond those who are at-risk for undesirable outcomes. Whereas protective factors mitigate or buffer the effects of a risk on an outcome [5], promotive factors can promote favorable outcomes regardless of the level (or presence) of risk; thus, their benefits may extend to a broader population. 

### 1.1. Child and Adolescent Flourishing 

Flourishing can be understood as the “combination of feeling good and functioning effectively,” [6], (p.837) and is recognized as an indicator of mental well-being in diverse child and adolescent populations [7]. More recently, general flourishing has been described as the amalgamation of positive emotion and a sense of self-achievement and accomplishment [8,9]. Characteristics of flourishing in children and adolescents include fostering positive relationships, participating in familial, social, and academic endeavors, exhibiting a sense of purpose, motivation, and self-fulfillment, as well as exhibiting positive strategies of coping and resiliency through adversity [10,11,12]. Conversely, the inability to develop or exercise these qualities is linked with adverse outcomes that may persist into adulthood. For example, poor coping skills, impulsivity, and the lack of motivation and interest in learning throughout childhood and adolescence may impede academic progress or success [11]. 

Flourishing is related to favorable health and well-being outcomes among children and adolescents. Previous research suggests that flourishing fosters the formation of healthy relationships and positive outcomes in mental, emotional, and physical health throughout adulthood [11,13]. Additionally, flourishing is inversely associated with depression, anxiety, panic attacks, physical pain, chronic disease, and suicidality [13,14,15]. Further, longitudinal research demonstrates that health and well-being are significantly better among those with high levels of flourishing [15] and predicts functioning and longevity in adults [13].

### 1.2. Family Resilience and Child and Adolescent Flourishing

Family resilience refers to the process that families undergo to cope with or adapt to demands and stress [16,17]. Because families play a pivotal role in child and adolescent health, development, and well-being [18,19], family resilience can promote flourishing in multiple ways. For example, family resilience can promote supportive relationships. Positive and supportive parent–child relationships are salient predictors of healthy child and adolescent outcomes [20].

In addition to directly supporting healthy development and well-being, supportive family relationships can also foster favorable outcomes such as flourishing by facilitating resilience among children and adolescents who face adversity [3]. For example, child psychopathology risks are significantly reduced among children whose mothers experienced depression if fathers engage in sensitive parenting practices characterized by displaying affection, support, resourcefulness, and encouragement [21]. Further, because family relationships can extend beyond parent–child relationships, supportive sibling relationships have also been found to buffer the effects of problematic relationships between parents on children. When exposed to intra-parental conflict, children who have positive relationships with their siblings demonstrate better adjustment than those without supportive relationships [22].

Family resilience can promote child and adolescent flourishing by buffering the impact of adversity. For example, a qualitative study of low-income, rural mothers reported strategies mothers would implement so that they could provide their children with a birthday celebration despite the economic challenges they faced [23]. Families can also mitigate the effect of adversity on their children by implementing strategies to help children and adolescents adjust to significant changes. In the global COVID-19 pandemic, children experienced major life disruptions due to quarantines. One study found that parents’ development of new home routines and emotional support were associated with lower levels of child internalizing and externalizing symptoms [24].

Family resilience can additionally influence flourishing among children and adolescents by modeling healthy behaviors. According to Social Learning Theory [25], children are constantly observing their parents and, over time, they can emulate the behaviors they observed. By witnessing how their parents and other family members respond to stressors and demands in healthy (e.g., seeking advice, relying on social support), children may also develop these skills, preparing them to respond to future adversity.

### 1.3. Neighborhood Physical and Social Environments and Child and Adolescent Flourishing

Neighborhood physical and social environments are vital contextual factors for the health, well-being, and development of children and adolescents [26,27] and can promote flourishing in various ways. Social cohesion, which refers to residents’ sense of belonging, safety, and acceptance within their community [28], plays a significant role in residents’ health, safety, and well-being. The idea of “group belonging” can benefit children and adolescents by encouraging them to explore and develop their identity and learn prosocial behaviors. Parents can also benefit from group belonging because of increased social support and the community’s monitoring of children and their activities [28,29,30]. 

Socially cohesive neighborhoods can directly protect and promote child and adolescent health, development, and flourishing. Social cohesion is associated with reduced stress and increased self-esteem, personal mastery, interpersonal autonomy, and mental health among adolescents regardless of urban or rural environmental classification [28]. Moreover, higher levels of neighborhood collective efficacy are inversely linked to adolescent depression and anxiety after controlling for socioeconomic status, household income, and sex of the child [31].

Socially cohesive neighborhoods can additionally foster safe environments for children and adolescents by activating community safeguarding among residents. Several studies indicate that adolescents who live and stay in violent, disadvantaged neighborhoods exhibited decreased levels of self-efficacy and increased levels of psychological distress compared to their counterparts who live or relocate to advantaged, less violent neighborhoods [26]. Equivalently, continued exposure to challenging environmental conditions (poverty, crime, violence, abuse, etc.) encumber developmental factors central to flourishing in adolescence [32]. 

Neighborhood social cohesion could also indirectly influence well-being and flourishing among children and adolescents by enabling family resilience. When parents have close ties with other neighborhood residents, they may draw on them for social and emotional support. Additionally, social cohesion can enhance parental health and well-being by facilitating health behaviors such as exercise [33]. These beneficial effects from social cohesion may then pass through parents to advantage their children.

Living in challenging environmental conditions is associated with lower levels of health and well-being among children and adolescents in the neighborhood [34,35]. However, the neighborhood built environment also plays a crucial role in adolescents development [36]. The relationships among the physical environment, social environment, and child and adolescent health and development are complex. The presence of positive physical neighborhood features like parks, roads, sidewalks, and recreation centers can promote child and adolescent health and well-being [37]. These positive physical features can affect adolescents directly by increasing physical activity, lowering stress, and reducing exposure to negative stimuli [38,39]. In addition, they can indirectly bolster child and adolescent well-being by creating opportunities for social interactions and social support for both children and families [39,40]. By providing areas for families to gather, physical neighborhood environments can facilitate relationship building among parents, thus providing parents with opportunities to develop additional social support and social capital.

A published systematic literature review found that while studies examining the relationship between the neighborhood built-environment and psychological processes are scarce, yet they are an important area of research [41]. Further, few child development studies have focused on the built environment [39], which could provide salient insights as to how these elements promote child and family resilience. Despite the risk of adverse outcomes associated with living in disadvantaged areas [34,35], children and adolescents living in such environments are more likely to demonstrate coping skills, a concept related to resilience [42,43]. Further, additional indirect paths may activate family processes, which could also bolster child and adolescent well-being.

### 1.4. Current Study 

We aim to expand the study of community and family promotive factors in child and adolescent well-being by focusing on how they might work directly and indirectly to foster flourishing among children and adolescents. Much of the previous resilience research in child and adolescent well-being tends to focus on the roles of protective and promotive factors as averting or attenuating an adverse outcome in the presence of risk(s). Conversely, less attention has focused on promotive models to understand how assets and resources can work together and lead to favorable child and adolescent outcomes regardless of risk. 

Few studies examining relationships between child and adolescent mental health and well-being have examined social and built environments together. The majority of studies reviewed tended to focus on problems or adverse outcomes instead of positive mental health outcomes, processes, or functioning [41]. Further, prior research has traditionally focused on children or adolescents instead of both groups. The current study aims to address some of these limitations by applying a promotive factors approach to understanding how social and built neighborhood environments can support familial resilience and, consequently, flourishing in children and adolescents.

Two complementary frameworks guided our study. First, the social-ecological model [44] posits that child and adolescent outcomes are dynamically shaped by surrounding social and structural environments at the inter-personal level, community level, and societal level. This model guided us to examine if community-level promotive factors, neighborhood social cohesion and a favorable neighborhood environment, were directly associated with child and adolescent flourishing and indirectly associated through an inter-personal level promotive factor, family resilience. In order to examine the potential of these possible direct and indirect promotive effects, we applied a promotive factors model [45], which focus on the main effects between promotive factors and outcomes, as opposed to interactional effects. To the best of our knowledge, no prior study has applied a promotive factors model to understand how community and family promotive factors may and directly and indirectly relate to child and adolescent flourishing through relationships with family resilience among a nationally representative sample of US children and adolescents. 

## 2. Materials and Methods

### 2.1. Participants and Procedure

Data were obtained from the publicly available combined 2018–2019 National Survey of Children’s Health (NSCH), a nationally representative survey of US children administered by the US Census Bureau and maintained by the Data Resource Center for Child and Adolescent Health (DRC) and the Child and Adolescent Health Measurement Initiative (CAMHI) [46]. In efforts to increase sample size, CAMHI combined the NSCH surveys from 2018 and 2019 [46]. Child development, physical and mental health, well-being, and social experiences and characteristics of children 0–17 years old questions were answered by the focal child’s parent or caretaker via online and paper surveys. Data collection for the 2018 NSCH occurred between June 2018 to January 2019, and data for the 2019 NSCH was collected between June 2019 and January 2020. The Child and Adolescent Health Measurement Initiative (CAMHI) combined the 2018 NSCH and 2019 NSCH into a single data file to enhance statistical power for researchers conducting analyses of the data because some variables had smaller sample sizes. The combined data file resulted in a total sample size of 59,963 (see [46] for detailed methodological information about the combined 2018–2019 NSCH data set). We selected an analytic subset of 43,213 children between the ages of 6–17 years old from the 2018–2019 combined NSCH data as these cases contained the ages of children and adolescents that were the focus of our study. We separated the analytic sample of children into two groups, (1) children between 6–11 years old and (2) adolescents aged 12–17 years old and ran the model separately for each group in the event relationships might differ by developmental timing (i.e., childhood vs. adolescence).

### 2.2. Measures

#### 2.2.1. Independent Variables

Neighborhood Social Cohesion. Neighborhood social cohesion characterizes residents’ perceptions of close-knit social ties and a sense of safety within their community. It was assessed as a latent variable using four items that described the neighborhood’s social environment, including perceptions of neighbors helping one another, watching out for children, the safety of children, and knowing where to go for help. Participants rated these items using a four-point scale (definitely disagree to definitely agree). The reliability coefficient for this scale demonstrated acceptable internal consistency for both the 6–11 and 12–17-year-old groups (alpha = 0.814 and 0.82, respectively).

Physical Neighborhood Environment. The latent variable neighborhood physical environment aimed to capture the conditions of the physical neighborhood environment and was assessed by four binary items that characterized physical environmental conditions (presence of walkways, parks/playgrounds, recreation centers, and libraries). Participants reported yes or no on the presence of these conditions; reliability analyses yielded acceptable internal consistency for both 6–11- and 12–17-year-olds (alpha = 0.734 and 0.749 respectively).

#### 2.2.2. Mediating Variable

Family resilience was assessed as a latent variable using four indicators, each measured on a four-point scale (none of the time, some of the time, most of the time, and all of the time). Participants were asked to rate their perceptions about the degree to which their family talked together, worked together when facing a problem, drew on strengths, and stayed hopeful. Reliability analyses demonstrate good internal consistency for the 6–11 and 12–17-year-old groups (alpha = 0.891 and 0.895, respectively).

#### 2.2.3. Dependent Variable

A latent variable for child and adolescent flourishing was assessed using three items that gauged participants’ perceptions of their child’s interest in and curiosity in learning new things, ability to complete the tasks they start, and ability to remain calm when challenged using a four-point scale (never to always). These items were developed for the NSCH to measure flourishing for children 6–17 years old [46]. Items were coded so that higher scores indicated greater flourishing. Scale reliability demonstrated acceptable thresholds for both children aged 6–11 and 12–17 years (alpha = 0.724 and 0.749, respectively).

#### 2.2.4. Covariates 

We controlled for several social determinants of health and health conditions that may affect child and adolescent flourishing. Economic hardship assessed participants’ perceptions of the frequency they could not afford family needs and was collapsed into two categories (never or rarely, and very often or somewhat often). Public assistance was measured as a binary variable using the receipt of at least one form of government assistance (Medicaid, food stamps, reduced lunches, subsidized housing). Child global health was measured on a five-point scale (excellent to poor) and was collapsed into three categories due to the small variability observed in the original five categories (by NSCH study personnel). Biological sex was measured as binary using males as the reference group. Race/ethnicity was measured using dummy variables for Black, Hispanic, Asian, and Multiracial, with White as the reference group. Chronic health condition was measured as a binary variable in which the child was reported to have at least one chronic health condition or none. 

### 2.3. Analytic Strategy

We employed structural equation modeling (SEM) to test the hypotheses such that neighborhood social cohesion and neighborhood physical environment directly predicted child and adolescent flourishing, and indirectly via family resilience. Covariates were regressed on the dependent variable to control for the possible effects of the children and adolescents’ biological sex, health, race/ethnicity, and family economic disadvantage and hardship. Identical mediation models were performed for each age group separately to examine whether relationships differed by age group. We used the maximum likelihood estimator (MLR) because it is a robust modal estimation method that can deal with non-normality and missing data. Measurement and structural models were evaluated using recommended thresholds for model fit non-significant chi-square of model fit (χ^2^_df_) [47,48], root mean square of error of approximation (RMSEA) < 0.06 [49], comparative fit index (CFI) and Tucker-Lewis Index (TLI) < 0.90 [48], and standardized root mean square residual (SRMR) < 0.08 [49]. Survey weights were applied to the analyses to account for the complex design of the 2018–2019 combined NSCH data. All SEM procedures were performed using Mplus version 8.3 [50].

## 3. Results

### 3.1. Descriptive Statistics 

Weighted descriptive statistics of the 6–11-year-old child samples demonstrated similar characteristics as those of the 12–17-year-old adolescent samples. Both samples for children and adolescents were nearly evenly divided between biological sex with males comprising 51.0% of the child sample, and 51.2% of the adolescent sample. The majority of children in both the child and adolescent samples identified as White, non-Hispanic (49.8% for both groups), and the majority resided with married parents (70.4% and 67.8%, respectively). Further, most of the children (58.6%) and adolescents (59.7%) resided in homes that were 200% of the US Federal Poverty Rate (sample characteristics are provided in Table 1).

### 3.2. Measurement Models

Measurement models for the latent variables were assessed via confirmatory factor analysis (CFA). Initial model fit indices for both the 6–11-year-old and 12–17-year-old groups did not meet recommended thresholds for several model fit indices (Table 2). Thus, we made minor model re-specifications which resulted in correlating item measurement errors between two indicators for each age group’s CFA; re-specifications were informed by evaluating the model modification indices and theory as to what may have contributed to the model misfit among these items (e.g., similar wording between items). Factor loadings for items assessing latent variables for child and adolescent models were statistically significant and ranged within acceptable thresholds (Table 3). 

### 3.3. Structural Models

#### 3.3.1. Children 

**Figure 1:**
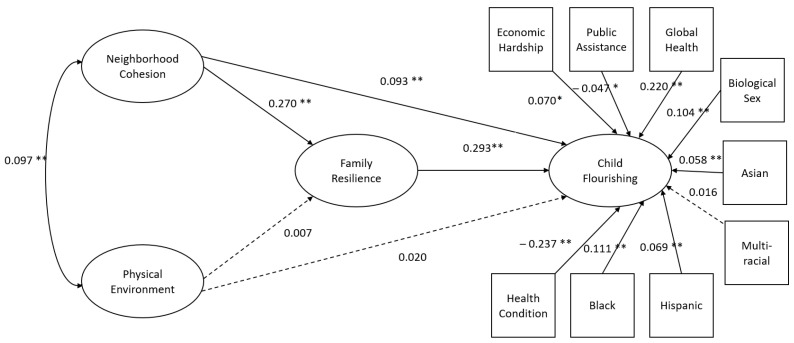
Child structural model. All model coefficients are standardized. * *p* < 0.05, ** *p* < 0.01. Solid lines represent statistically significant relationships. Dashed lines represent non-statistically significant relationships.

The structural model for children aged 6–11 years yielded adequate model fit with exception to the model chi-square (Table 4), which can be sensitive to a large sample size. The model chi-square can be sensitive to large sample sizes [51], thus, multiple indices were used to assess fit. Statistically significant structural paths indicated that the physical environment was not a significant predictor of either family resilience or child flourishing (Figure 1). Conversely, neighborhood social cohesion is directly associated with child flourishing (β = 0.093, *p* < 0.01). The indirect relationship between neighborhood social cohesion and child flourishing, in which neighborhood social cohesion is associated with family resilience (β = 0.270, *p* < 0.01) and family resilience associated with child flourishing (β = 0.293, *p* < 0.01), was also statistically significant. Significant covariates included inverse relationships between child flourishing and economic hardship (β = −0.070, *p* < 0.01), public assistance (β = −0.047, *p* < 0.01), and having at least one chronic health condition (β = −0.237, *p* < 0.01). On the contrary, higher levels of global health (β = 0.220, *p* < 0.01), male biological sex (β = 0.104, *p* < 0.01), and identifying as Black, Asian, or Hispanic (β = 0.111, 0.069, 0.058, *p* < 0.05, respectively) were associated with higher levels of flourishing. 

#### 3.3.2. Adolescents 

**Figure 2:**
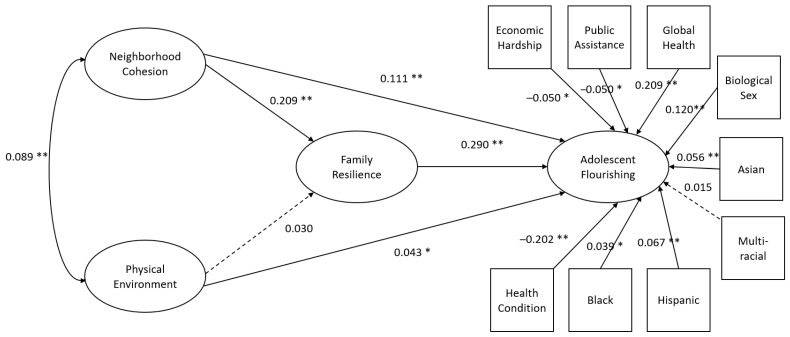
Adolescent structural model. All model coefficients are standardized. * *p* < 0.05, ** *p* < 0.01. Solid lines represent statistically significant relationships. Dashed lines represent non-statistically significant relationships.

Model fit indices, with the exception of model chi-square, suggested acceptable fit (Table 4). Significant structural paths identified that physical environment, neighborhood social cohesion, and family resilience were positively associated with higher levels of adolescent flourishing (Figure 2). As observed in the child model, indirect effects were statistically significant (*p* < 0.05). The relationship between neighborhood social cohesion and adolescent flourishing was, in part, accounted for by family resilience. Given the positive relationships between variables, as neighborhood social cohesion increased, family resilience and adolescent flourishing also increased. Unlike the child model, the physical environment was directly associated with higher levels of adolescent flourishing, but it was not associated with nor mediated by family resilience. Relationships between adolescent flourishing and model covariates paralleled the child structural model; economic hardship (β = −0.050, *p* < 0.01), public assistance (β = −0.050, *p* < 0.01), and having at least one chronic health condition (β = −0.202, *p* < 0.01) were negatively associated with adolescent flourishing whereas male biological sex (β = 0.120, *p* < 0.01), global health (β = 0.209, *p* < 0.01) identifying as Black, Asian, or Hispanic (β = 0.039, 0.056, 0.067, *p* < 0.05, respectively) were associated with higher levels of flourishing. 

## 4. Discussion

Identifying elements that bolster flourishing within the child and adolescent population are imperative to promote health and success later in life. Moreover, a greater understanding of the individual, familial, and environmental predictors of flourishing will further inform and increase the effectiveness of future programs and policies in schools, communities, and adolescent service agencies. Therefore, research must begin to unravel the socioecological influence on flourishing and provide continued support for the growth, development, and resilience of today’s adolescents. Resilience studies can benefit by expanding beyond investigating how protective factors avert or mitigate risks. Specifically, investigations examining how promotive factors can work with or through other protective and promotive factors offer critical insights into how children and adolescents can benefit from multiple sources of resilience. The current study was guided by a social ecological framework to examine the latter by applying a promotive factors model to investigate how community-level and family-level assets and resources might work in tandem to promote child and adolescent flourishing. 

### 4.1. Family Resilience 

At the family-level, our findings demonstrated that higher levels of family resilience were related to higher levels of flourishing among children and adolescents while statistically controlling for multiple social determinants of health (e.g., SES, race/ethnicity). Family resilience may promote flourishing by fostering nurturing social environments that provide children and adolescents with support, hope, and encouragement. Additionally, resilience processes within families can enable flourishing by helping children and adolescents to develop healthy coping and problem-solving skills via parental modeling, direction, and experience. Young children may witness how their families collectively work to resolve problems while also encouraging one another and instilling hope. Family resilience could promote flourishing among adolescents by providing adolescents with direct problem-solving experiences. For instance, because adolescence is a developmental time when adolescents begin to build autonomy, parents help their adolescent children’s responses to adversity by coaching them and incorporating them into family problem-solving decisions. 

### 4.2. Physical and Social Neighborhood Environments

At the community level, for both children and adolescents, living in a socially cohesive neighborhood was associated with higher levels of flourishing. Neighborhood social cohesion may foster a sense of security for children and parents. Additionally, when parents have close ties with neighbors, they may be more willing to let their children interact with other neighborhood children [52]. Among adolescents, a socially cohesive neighborhood can offer parents additional monitoring and supervision [53]. Similar to children, parents/caretakers who assess their communities to be safe and supportive may be more willing to permit their adolescents to get outside and engage with others in the community [52]. 

Neighborhood physical environment was significantly related to higher levels of flourishing among adolescents but not children. This finding may be due to the increased independence and autonomy that often comes with adolescence. Parents typically place more trust in adolescents to safely navigate their neighborhoods as they age. As a result, adolescents are more likely to spend time in their neighborhood than younger children and are more likely to be affected by their physical environment [54]. 

### 4.3. Indirect Relationships

Given that the social ecological model suggests that the surrounding social and structural environments can influence child and adolescent outcomes, we examined if community-level promotive factors and a family-level promotive factor might work in tandem to associate with child and adolescent flourishing. Indirect relationships between neighborhood social cohesion, family resilience, and flourishing were significant for adolescents but not children. Because the child model sample included children between the ages of 6–11 years old, it may be that their parents were less willing to allow their children to spend much time around the neighborhood without their supervision. Further, it is also possible that parents of younger children may be more conservative when assessing their family’s neighborhood and physical environment; parental assessments of neighborhood social environments have been linked with children’s independent mobility [55]. More recent research supports that parents’ permission for their children’s independent mobility is declining [56,57]; future research should examine how this trend may impact relationships between social cohesion and child flourishing.

Among families with adolescent children, living in socially cohesive neighborhoods might contribute to their resilience in multiple ways. First, when parents/caretakers have supportive connections with others in their community, they have access to additional emotional and social support outside of their family. Raising adolescents entails both rewarding and challenging experiences—having neighbors who can provide emotional support may be beneficial for the parent/caretaker’s well-being and thus explain the indirect relationship between neighborhood social cohesion and adolescent flourishing. Additionally, trusting relationships between parents and neighbors could facilitate seeking help with material support if needed. Parents who believe they can draw on this support if the need arises may feel more confident in their parenting role, thus bolstering family resilience. Further, communities with high levels of social cohesion may be more likely to watch out for the children and adolescents within the neighborhood, providing higher levels of safety. As a result, parents may feel reduced stress in their caretaking tasks because they feel that the neighborhood, in general, is a place where their child will be protected. Fourth, social cohesion may contribute to the ability of children and adolescents to engage within their neighborhood and make connections with other children and adults living in the neighborhood. Safe, stable, and nurturing relationships are critical for healthy child development [58]. Taking all of these together, we suggest that neighborhood social cohesion increases positive outcomes for children and parents alike, resulting in improved functioning of the family unit as a whole and the interactions within. Improved family functioning, in turn, contributes to positive outcomes in children and adolescents. 

Contrary to our hypothesis and prior literature, indirect effects between physical neighborhood environments, family resilience, and flourishing were not statistically significant for either children or adolescents. In a previous study that used data from the 2007 NSCH, family functioning was found to mediate the relationship between neighborhood physical resources and global child health among children aged 6–17 years old [59]. There is overlap between family resilience and family function, but the latter captures other processes not related to resilience, including parenting stress. Therefore, the neighborhood physical environment may relate more to a constellation of family processes than resilience. Additionally, our null finding may also be due to the limitation that the measure for neighborhood physical environment only asked whether amenities like parks and recreation centers were present, not if the families were using them or how they perceived their quality. Future research is warranted to examine further what might hinder relationships between physical neighborhood environments and family resilience.

### 4.4. Limitations

The current study’s findings should be considered with the following limitations. First, as with all secondary data analyses, the original data were not designed to address the research questions of the current study. We believe that the available data offered suitable measures of the constructs of interests, and the models still offer important insights. Second, given the cross-sectional nature of the data, temporal support for causality is not possible. However, we drew from theory and prior literature, such as other studies that have tested indirect effects using cross-sectional data from the NSCH (e.g., [60,61,62], to support the hypothesized relationships. Future studies should examine the relationships between neighborhood social and physical environments, family resilience, and child and adolescent flourishing over time. As with any study examining the neighborhood-built environment, it is necessary to discuss the role of socioeconomic status in shaping the built environment. Typically, higher socioeconomic status neighborhoods are more walkable and have amenities such as parks [63]. Hence, we were careful to select covariates for the dependent variable that could control for such effects, including economic hardship and public assistance receipt. Despite these limitations, we believe that the current study offers important insights in the arena of child and adolescent flourishing, and these data allowed us to make estimates of the general population of non-institutionalized children and adolescents residing in the US. 

## 5. Conclusions

Children and adolescents thrive in social and built environments that support their health and well-being. While these young persons spend most of their time in the family environment, the sociological model suggests that family environments are influenced by additional surrounding environments (e.g., neighborhoods, communities). Hence, individual family members are also affected by these extra-familial environments. 

We found that above and beyond the effects of chronic health problems, economic disadvantage, race/ethnicity, and biological sex, child and adolescent flourishing was bolstered by socially cohesive neighborhoods and family resilience. Further, while the neighborhood physical environment did not associate with family resilience for children and adolescents, it directly associated with higher levels of flourishing for adolescents, though not children. Understanding what strengthens and enhances the protective and promotive factors of child and adolescent flourishing is critical for designing prevention, intervention, and policy efforts aimed at realizing optimal health and well-being for these vulnerable members of society. Such efforts should consider incorporating community social cohesion and family resilience into programs and other endeavors that aim to promote child and adolescent well-being. Tapping into these promotive factors may not only bolster new or existing programs, but they honor and recognize strengths that may already be present in communities and families. Resilience research is further enriched by expanding its scope to include models that examine how promotive factors contribute to child well-being and development, despite the level of risk.

## Figures and Tables

**Table 1:** Sample characteristics.

Characteristic	Children 6–11 Years Old		Adolescents 12–17 Years Old	
	Unweighted Frequency	Weighted Percent	Unweighted Frequency	Weighted Percent
**Age (years)**				
6–8	8484	48.3%		
9–11	9912	51.7%		
12–14			11,124	50.4%
15–17			13,693	49.6%
**Child’s biological sex**				
Male	9571	51.0%	12,956	51.2%
Female	8825	49.0%	11,861	48.8%
**Race/ethnicity**				
White (non-Hispanic)	12,514	49.8%	17,501	49.8%
Black (non-Hispanic)	1251	13.9%	1639	14.0%
Asian (non-Hispanic)	870	4.7%	1218	4.6%
Multiple race (non-Hispanic)	1493	6.4%	1630	5.2%
Hispanic (any race)	2268	25.2%	2829	26.3%
**Child has at least one chronic health condition**	8545	43.0%	12,978	47.3%
**FPL of household**				
0–99%	2278	19.6%	2675	18.9%
100–199%	3140	21.8%	3917	21.4%
200% or greater	12,978	58.6%	18,225	59.7%
**Primary parent/caretaker**				
Employed	13,758	69.4%	19,127	70.8%
Married	13,640	70.4%	18,367	67.8%
Divorced/separated	1968	10.4%	3499	15.6%
Never married	1085	8.1%	1011	6.4%
**Child’s health is excellent or very good**	16,912	90.1%	22,189	87.4%
**Family economic hardship frequency**				
Very or somewhat often	2466	16.2%	3252	16.0%
Never or rarely	15,616	83.8%	21,130	84.0%

**Table 2:** Measurement models.

Model	Fit Index	Child Model	Adolescent Model
Initial	χ^2^_df_	χ^2^_84_ = 989.702 **	χ^2^_84_ = 839.470 **
	RMSEA	0.024 (0.023–0.026)	0.019 (0.018–0.020)
	CFI	0.951	0.963
	TLI	0.938	0.9954
	SRMR	0.029	0.079
Modified	χ^2^_df_	χ^2^_82_ = 488.463 **	χ^2^_83_ = 557.513 **
	RMSEA	0.016 (0.015–0.018)	0.015 (0.014–0.016)
	CFI	0.978	0.977
	TLI	0.972	0.971
	SRMR	0.024	0.032

** *p* < 0.01; df = degrees of freedom; RMSEA = root mean square of error of approximation; CFI = comparative fit index; TLI = Tucker-Lewis Index; SRMR = standardized root mean square residual.

**Table 3:** Standardized factor loadings (λ) for the final measurement models.

Latent Variable	Item	Child Model λ	Adolescent Model λ
Neighborhood Cohesion			
	People in neighborhood help each other out	0.838 **	0.856 **
	People In neighborhood watch out for other’s children	0.822 **	0.835 **
	Child is safe in neighborhood	0.610 **	0.601 **
	Know where to go for help in neighborhood	0.660 **	0.627 **
Physical Environment			
	Neighborhood has sidewalks or walking paths	0.483 **	0.538 **
	Neighborhood has park or playground	0.679 **	0.720 **
	Neighborhood has recreation center	0.636 **	0.655 **
	Neighborhood has library or bookmobile	0.687 **	0.655 **
Family Resilience			
	Family talks together when facing problems	0.773 **	0.881 **
	Family works together when facing problems	0.824 **	0.942 **
	Family draws on strengths when facing problems	0.890 **	0.786 **
	Family stays hopeful when facing problems	0.703 **	0.667 **
Flourishing			
	Child shows interest and curiosity in learning new things	0.584 **	0.632 **
	Child works to finish the tasks they start	0.820 **	0.798 **
	Child stays calm and in control when faced with a challenge	0.685 **	0.690 **

Items have been paraphrased for purposes of brevity. ** *p* < 0.01.

**Table 4:** Structural model fit statistics.

Fit Index	Child Model	Adolescent Model
χ^2^_df_	χ^2^_208_ = 1526.939 **	χ^2^_209_ = 1781.037 **
RMSEA	0.019 (0.018–0.020)	0.018 (0.017–0.019)
CFI	0.939	0.930
TLI	0.929	0.920
SRMR	0.054	0.060

** *p* < 0.01; df = degrees of freedom; RMSEA = root mean square of error of approximation; CFI = comparative fit index; TLI = Tucker-Lewis Index; SRMR = standardized root mean square residual.

## Data Availability

Data used in this project originated from the National Survey of Children’s health, which is supported by the Health Resources and Services Administration (HRSA) of the U.S. Department of Health and Humans Services (HHS) under grant number U59MC27866, National Maternal, and Child Health Data Resource Initiative, $4.5 M. This information or content and conclusions are those of the author and should not be construed as the official position or policy of, nor should any endorsements be inferred by HRSA, HHS, or the U.S. Government. Data are publically available at: https://www.childhealthdata.org/help/dataset (accessed on 16 February 2022).
